# Genomic Prediction of Resistance to Pasteurellosis in Gilthead Sea Bream (*Sparus aurata*) Using 2b-RAD Sequencing

**DOI:** 10.1534/g3.116.035220

**Published:** 2016-09-20

**Authors:** Christos Palaiokostas, Serena Ferraresso, Rafaella Franch, Ross D. Houston, Luca Bargelloni

**Affiliations:** * The Roslin Institute and Royal (Dick) School of Veterinary Studies, University of Edinburgh, Midlothian, EH25 9RG Scotland, United Kingdom; † Department of Comparative Biomedicine and Food Science, University of Padova, 35020 Legnaro, Italy

**Keywords:** GenPred, Shared Data Resources, Genomic Selection, 2b-RAD, Aquaculture, Breeding, High-Throughput sequencing

## Abstract

Gilthead sea bream (*Sparus aurata*) is a species of paramount importance to the Mediterranean aquaculture industry, with an annual production exceeding 140,000 metric tons. Pasteurellosis due to the Gram-negative bacterium *Photobacterium damselae* subsp. *piscicida* (Phdp) causes significant mortality, especially during larval and juvenile stages, and poses a serious threat to bream production. Selective breeding for improved resistance to pasteurellosis is a promising avenue for disease control, and the use of genetic markers to predict breeding values can improve the accuracy of selection, and allow accurate calculation of estimated breeding values of nonchallenged animals. In the current study, a population of 825 sea bream juveniles, originating from a factorial cross between 67 broodfish (32 sires, 35 dams), were challenged by 30 min immersion with 1 × 10^5^ CFU virulent Phdp. Mortalities and survivors were recorded and sampled for genotyping by sequencing. The restriction-site associated DNA sequencing approach, 2b-RAD, was used to generate genome-wide single nucleotide polymorphism (SNP) genotypes for all samples. A high-density linkage map containing 12,085 SNPs grouped into 24 linkage groups (consistent with the karyotype) was constructed. The heritability of surviving days (censored data) was 0.22 (95% highest density interval: 0.11–0.36) and 0.28 (95% highest density interval: 0.17–0.4) using the pedigree and the genomic relationship matrix respectively. A genome-wide association study did not reveal individual SNPs significantly associated with resistance at a genome-wide significance level. Genomic prediction approaches were tested to investigate the potential of the SNPs obtained by 2b-RAD for estimating breeding values for resistance. The accuracy of the genomic prediction models (*r* = 0.38–0.46) outperformed the traditional BLUP approach based on pedigree records (*r* = 0.30). Overall results suggest that major quantitative trait loci affecting resistance to pasteurellosis were not present in this population, but highlight the effectiveness of 2b-RAD genotyping by sequencing for genomic selection in a mass spawning fish species.

Infectious diseases are a major threat to the profitability, sustainability, and welfare status of farmed fish production (Yáñez *et al.* 2014). Gilthead sea bream (*Sparus aurata*) is one of the most important farmed fish in Mediterranean countries, with an annual production of ∼146,000 metric tons (Federation of European Aquaculture Producers 2014). Pasteurellosis due to *Photobacterium damselae* subsp. *piscicida* (Phdp) is one of the primary disease problems faced by the sea bream aquaculture industry. High levels of mortality (∼90–100%) are frequently observed, especially in periods where water temperature rises above 18°, with larvae and juveniles being most susceptible (Noya *et al.* 1995; Magarinos *et al.* 2001).

Selection for improved genetic resistance in aquaculture breeding schemes is a valuable tool to help prevent or reduce disease outbreaks, especially where effective therapeutic agents or vaccines are lacking (Bishop and Woolliams 2014). Moderate to high heritabilities have been estimated for resistance to many common diseases, indicating that rapid genetic progress can be made through selective breeding (Ødegård *et al.* 2011). In addition, recent technological advances in genome-wide sequencing and genotyping technology offer the potential of deriving more accurate estimated breeding values (EBVs) for individual selection candidates, as compared to the classical breeding approach where breeding values are typically estimated at a family level (Goddard and Hayes 2009). The application of genomic data to breeding is particularly valuable for disease resistance, which is typically expensive or impossible to measure on the selection candidates themselves. While marker-assisted selection for major disease resistance loci has been well documented in Atlantic salmon (*Salmo salar*) breeding programs (Houston *et al.* 2008, 2010; Moen *et al.* 2009), few successful examples exist for other farmed finfish species. Genomic prediction uses genome-wide markers to estimate breeding values, and can deliver significant improvements in selection accuracy compared to traditional pedigree-based approaches, even for traits with a polygenic architecture (Odegård *et al.* 2014; Tsai *et al.* 2015, 2016; Vallejo *et al.* 2016).

Previous studies into the genetic resistance to pasteurellosis in sea bream have detected resistance quantitative trait loci (QTL) using microsatellite markers (Antonello *et al.* 2009; Massault *et al.* 2010). However, these studies were restricted by the low resolution of microsatellite markers, as compared to the high-density single nucleotide polymorphism (SNP) genotypes offered by SNP arrays (*e.g.*, Houston *et al.* 2014) or genotyping-by-sequencing approaches (Davey *et al.* 2011). Restriction-site associated DNA (RAD) sequencing is a reduced representation high-throughput sequencing technique for the concurrent detection and genotyping of SNP markers in multiplexed samples with a unique nucleotide barcode (Baird *et al.* 2008). RAD sequencing and similar genotyping-by-sequencing techniques rely on digestion of the genomic DNA with a restriction enzyme, and subsequent high-depth sequencing of the flanking regions. These techniques have been applied in several studies of aquaculture species to generate high-density linkage maps (*e.g.*, Palaiokostas *et al.* 2013a,b; Gonen *et al.* 2014; Palaiokostas *et al.* 2015) and perform genome-wide association studies (GWAS) in a cost-efficient manner (Campbell *et al.* 2014). A flexible and easily streamlined variation of RAD sequencing, named 2b-RAD sequencing, utilizes type IIB restriction enzymes to cleave genomic DNA upstream and downstream of the target site (Wang *et al.* 2012). In theory, 2b-RAD samples all the endonuclease recognition sites for sequencing, circumventing potential biases that may result from the size selection step in the original RAD protocol (Puritz *et al.* 2014). 2b-RAD data have also been applied for genetics studies in aquaculture species, for example to test genomic prediction in a limited number of Yesso scallop (*Patinopecten yessoensis)* families (Dou *et al.* 2016).

In this study, we used 2b-RAD sequencing to identify and genotype genome-wide SNPs in juvenile sea bream challenged with virulent Phdp bacteria, and recorded for survival time. A high-density SNP linkage map was constructed and a GWAS was performed to test the association between individual loci and resistance to pasteurellosis. Finally, genomic prediction of resistance was tested using several genomic selection models and marker densities to evaluate its potential in selection for improved resistance to pasteurellosis in sea bream.

## Materials and Methods

### Sample collection and preparation

The experimental population used in the present experiment was part of a larger group of juvenile sea bream that were subjected to an experimental challenge with Phdp to estimate heritability of disease resistance, as reported in Antonello *et al.* (2009). Fish were provided by the fish farm Valle Ca’ Zuliani (Monfalcone, Italy). All broodstock fish were originally sampled from wild populations. Fertilized eggs were collected on the same day (year 2006), from natural mass spawning events occurring in four different broodstock tanks; therefore all fish had approximately the same age. Each broodstock tank contained 50–60 fish with a sex ratio 3:1 females to males. Approximately 10,000 eggs were collected, pooled, and kept in a separate tank without any size sorting until 110 d old.

All fish were then transferred to the Istituto Zooprofilattico Sperimentale delle Venezie (Legnaro, Italy) for the experimental challenge. Fish were divided into two aerated tanks (A and B), each with 800 liter of recirculating seawater (salinity 35 ppt). Water temperature was maintained at 19°. After 1 wk of acclimation, fish were experimentally infected with a highly virulent strain of Phdp (strain 249/ittio 99), as described in Antonello *et al.* (2009). Mortality was monitored daily for 19 d (Supplemental Material, Table S1). Mortality levels were nearly identical for both tanks, and only fish from tank A were included in the current study. Fish used in the challenge originated from 67 broodfish (32 sires, 35 dams). As already described in Antonello *et al.* (2009) and Massault *et al.* (2011), parentage analysis was carried out using a panel of nine microsatellite loci (Table S2).

### 2b-RAD library preparation and sequencing

A total of 892 2b-RAD libraries (67 parents and 825 juveniles) were constructed by following the protocol reported by Wang *et al.* (2012), with some modifications (Pecoraro *et al.* 2016). Template DNA for each individual (500 ng) was digested in 6 μl reaction volume using 1 U AlfI (Thermo Fisher Scientific) at 37° for 1 hr, followed by enzyme heat inactivation at 65° for 20 min. The ligation reaction was performed by combining 5 μl of digested DNA with 20 μl of a ligation master mix containing 0.4 μM each of two library-specific adaptors with fully degenerate cohesive ends (5′ -NN- 3′), 0.2 mM adenosine 5′-triphosphate (New England Biolabs), and 1000 U T4 DNA ligase (CABRU, Arcore, Italy). Ligation was carried out at 16° for 3 hr, with subsequent heat inactivation for 10 min at 65°.

Sample-specific barcodes were designed through a Barcode Generator program (http://comailab.genomecenter.ucdavis.edu/index.php/Barcode_generator). PCR reactions (50 μl) were prepared containing 12 μl of ligated DNA product, 0.2 μM of each primer, 0.3 mM dNTP, 5× Phusion HF buffer, and 2 U Phusion high-fidelity DNA polymerase (New England Biolabs). Each library was PCR amplified using the following conditions: 13 cycles of 95° for 5 sec, 60° for 20 sec, and 72° for 5 sec.

Adaptor and primer sequences were those reported in Wang *et al.* (2012). PCR products were purified using the SPRIselect purification kit (Beckman Coulter, Pasadena, CA) and quantified through a Qubit 2.0 Fluorometer (Invitrogen). The quality of all amplicon libraries was checked at 1.8% agarose gel. Additionally, the quality of 10% of randomly selected libraries was also assessed by running them on an Agilent 2100 Bioanalyzer.

Individual libraries were pooled into equimolar amounts by adopting two different multiplexing strategies for parents (24 libraries per pool) and offspring (48 libraries per pool). The quality of each pool was verified on Agilent 2100 Bioanalyzer. Finally, pooled libraries were sequenced on an Illumina HiSeq2500 platform (Illumina, San Diego, CA) using 50 base single-end sequencing (v3 chemistry).

### Genotyping RAD alleles

Quality and adapters trimming of sequenced reads were performed by running a customized script (Pauletto *et al.* 2016; Pecoraro *et al.* 2016), obtaining 34-bp long fragments. SNP calling was performed using STACKS v1.23 (Catchen *et al.* 2013).

For each family/cross, individual genotypes were constructed using components of the STACKS pipeline as follows: (i) for each individual, *ustacks* program was employed for building loci from all quality control (QC)-passed reads using parameters *-m 10 -M 2 -N 3* for parents and *-m 5 -M 2 -N 3* for offspring, (ii) a catalog of loci unique for all families/crosses was constructed by using all parents’ reads on *cstacks* program, then (iii) each set of parents/offspring per cross was matched separately against such a catalog (*sstacks* program), followed by genotype assignment by setting the following parameters on the *genotypes* program: *-c–min_hom_sequations 7–max_het_sequations 0.05*.

Unique tags created by STACKS were mapped against a draft assembly of *S. aurata* genome (L. Bargelloni, personal communication). Mapping analysis was carried out by means of CLC Genomic Workbench 7.5, with stringent criteria (length fraction = 0.9, similarity fraction = 0.9, nonspecific match handling = ignore).

### Linkage map construction

Linkage map construction was performed using Lep-Map v2 (Rastas *et al.* 2013). QC was performed for each full sibling family by excluding SNPs with minor allele frequency <0.05 and those deviating from expected Mendelian segregation (*P* < 0.001). Linkage groups were formed using a minimum LOD threshold value of 8 in the *SeparateChromosomes* module, allowing a maximum distance between consecutive SNPs of 50 cM. Marker order within each linkage group was performed using the *OrderMarkers* module, where the likelihood of marker order is computed by using a hidden Markov model (Rastas *et al.* 2013). Map distances were calculated in centimorgans, using the Kosambi mapping function.

### Trait definition and heritability estimation

Heritability of surviving days was estimated with the R/BGLR software (Pérez and de Los Campos 2014), using both the pedigree-based and the genomic relationship matrix.

The animal model was applied:

y=μ+Zu+e,

where **y** is the vector of recorded phenotypes (days to death; animals surviving at the end of the experiment treated as missing values sampled from corresponding truncated normal distribution with the resulting values being ≥20), ***μ*** is the vector of the intercept, **Z** is the incidence matrix relating phenotypes with the random animal effects, **u** is the vector of animal effects ∼*N*(0, **A***σ*_g_^2^) with either **A** corresponding to the pedigree-based relationship matrix or **G** corresponding to the genomic relationship matrix, and *σ*_g_^2^ corresponding to the additive genetic variance. Finally, **e** is the vector of residuals. The **G** matrix was estimated according to VanRaden (2008) using the *kin* function of the R/synbreed package (Wimmer *et al.* 2012; File S1). The additive genetic variance was estimated by applying Markov Chain Monte Carlo (MCMC) algorithm, using a prior that followed an inverse scaled x2 distribution (df = 5). The MCMC used 10-Million iterations, first 10% of which were discarded, and values were stored every 1000 iterations thereafter. Convergence of the resulting posterior distribution was assessed both visually (inspecting the resulting MCMC plots) and analytically with Geweke’s diagnostic using R/boa v1.1.7 (Smith 2007).

Heritability for the number of surviving days was estimated using the following formula:

$$h^{2}=\frac{\sigma_{g}^{2}}{\sigma_{g}^{2}+\sigma_{e}^{2}},$$

where $\sigma_{g}^{2}$ estimated additive genetic variance and $\sigma_{e}^{2}$ the residual variance.

### GWAS

To test the association between individual SNPs and resistance to pasteurellosis (measured as surviving days), a GWAS was performed using R/rrBLUP (Endelman 2011). The mixed model applied was based on Yu *et al.* (2006) and had the following format:

y=Xα+Zu+e,

where **y** is the vector of the phenotypes (surviving days or overall survival), ***α*** is the vector of unknown marker effects, **u** is the vector of animal random effects ∼*N*(0, **G$\sigma_{g}^{2}$**), and **e** is the vector of residuals. The matrix **G** represents the genomic relationship matrix as described above, and $\sigma_{g}^{2}$ is the additive genetic variance estimated using REML. **X** and **Z** are incidence matrices relating **y** to ***α*** and **u**, respectively. According to the above model, additive SNP effects are treated as fixed effects, with the inclusion of the random animal effect to decrease spurious associations due to (genomic) relationships between the animals (Yu *et al.* 2006). The genome-wide significance threshold for the estimated additive SNP effects was calculated using a Bonferroni correction (0.05/*N*), where *N* represents the number of QC-filtered SNPs across the entire genome.

### Genomic prediction

A genomic prediction approach was conducted to quantify the accuracy of the breeding values estimated using the SNP markers to predict the phenotypic trait values (surviving days). SNPs with >15% missing genotypes were removed in order to minimize impact of imputed genotypes, since used software cannot handle missing genotypes. Missing values of the remaining SNPs were imputed using R/synbreed (Wimmer *et al.* 2012). Genomic breeding values were estimated using rrBLUP, BayesA, BayesB (Meuwissen *et al.* 2001), and BayesC (Habier *et al.* 2011) models using the R/BGLR (Pérez and de Los Campos 2014) software. The above models differ in regard to the prior distribution of the marker effects. Briefly, rrBLUP induces homogenous shrinkage across markers by using the Gaussian distribution, while in BayesA the usage of a scaled-*t* distribution induces marker size effect shrinkage, allowing for variable marker effect sizes. Models BayesB and BayesC also perform variable selection, with the difference between the two being the usage of a scaled-*t* or a Gaussian prior density, respectively (Meuwissen *et al.* 2001; de los Campos *et al.* 2013). To compare the accuracy of genomic EBVs (GEBVs) to the pedigree-based EBVs, pedigree-based BLUP (PBLUP; Henderson 1975) was applied to calculate breeding values using the same software.

The general form of the fitted models was the following:

y=η+ε,

where **y** is the vector of phenotypic records, ***η*** is the linear predictor, and **e** is the vector of residuals.

The linear predictor, ***η***, in the case of rrBLUP, BayesA, BayesB, and BayesC, had the following general form:

$$\eta =1\mu +X_{1}\beta_{1},$$

where ***μ*** is the intercept, **X_1_** is the design matrix relating the phenotypes to the markers, and ***β*_1_** is the vector of marker effects with corresponding priors depending on the model used. Marker coding followed the format where the heterozygotes were coded as 1 and the two alternate homozygotes as 0 and 2.

The linear predictor, ***η***, in the case of pedigree BLUP, had the following form:

η=1μ+u,

where **u** is the animal random effect vector ∼*N*(*0*, **A***$\sigma_{g}^{2}$*) with the matrix **A** representing the pedigree estimated relationship matrix. The parameters of the above models were estimated through MCMC (110,000 iterations; burn-in: 10,000; thin: 100).

Assessment of the accuracy of breeding value predictions was conducted according to the following procedure. The data set was randomly split into a training set (*n* = 578 animals) and a validation set (*n* = 200). The above was repeated 100 times, with the obtained prediction accuracies being adjusted for the trait heritability for each tested model. The GEBVs for each replicate of the validation data set were estimated as:

GEBV=Xu,

where **X** is the incidence matrix relating GEBV with SNP genotypes and **u** is the vector of estimated SNP effects from the corresponding training data set.

The accuracy of the estimated GEBV was approximated as:

r=(GEBV,y)/h,

where **y** is the vector of recorded phenotypes and *h* is the square root of the heritability. In all tested scenarios, the heritability estimated using the genomic relationship matrix was used. Reported accuracies for each tested model refer to the mean accuracy of the above-mentioned 100 replicates of validation data sets.

In order to test the predictive ability of varying SNP densities, the above procedure was followed using (i) SNPs spaced >1 cM apart (2614 SNPs) or (ii) SNPs spaced >5 cM apart (705 SNPs) on the linkage map.

### Data availability

Raw reads were deposited in the European Bioinformatics Institute (EBI) repository under project ID SRP081498. Table S1 contains the phenotypic data. Table S2 contains the pedigree. Table S3 and Table S4 contain summaries of obtained reads for parental and offspring samples respectively. Table S5 contains the location of the linkage map SNPs. Table S6 contains the genotypic data. File S1 contains the R script used for genomic prediction.

## Results

### Disease challenge

**Figure 1:**
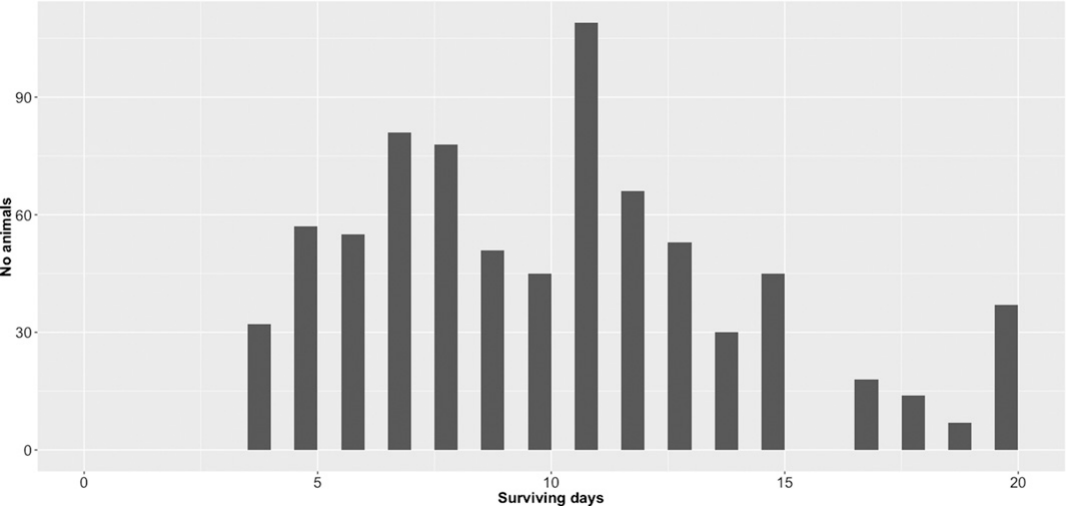
Distribution of surviving days across disease challenge. Frequency of mortalities per day during 19 d of challenge. Survivors were assigned a value of 20.

The challenged population consisted of 75 full sibling families, with a mean family size of 10, originating from a factorial cross between 67 broodfish (32 sires, 35 dams). The largest full-sib family consisted of 114 animals, while the smallest had only two animals (three full-sib families). The overall survival at the end of the pasteurellosis challenge was 4.7%. Observed mortality levels showed three distinct peaks on day 7 (10.4% loss), day 11 (14% loss), and day 15 (5.7% loss), followed by a steady reduction in daily mortality rate (Figure 1).

### Genotyping RAD alleles

The mean number of raw reads was 8.76 million and 4.51 million, while the number of reads passing QC was 7.74 million (88%) and 3.69 million (81%) for parents (Table S3) and offspring (Table S4), respectively. The STACKS catalog consisted of 202,598 unique 2b-RAD loci, of which 73,876 contained at least one SNP in the parents (EBI repository SRP081498). To confirm the identity of loci created by STACKS, the 202,598 tags of the catalog were mapped against an *S. aurata* draft reference genome assembly (L. Bargelloni, unpublished data). A high percentage of 2b-RAD loci were successfully mapped, with 93.5% of tags showing a unique match to the reference genome. In order to maximize the number of informative SNPs and minimize the amount of missing or erroneous data, RAD-tags that were retrieved in at least 75% of the samples, and that carried only one or two SNPs were retained. A total of 48 animals with missing data >30% were excluded from subsequent analysis. A total of 21,974 putative SNPs were finally used for construction of the genetic map from 777 disease challenged offspring (genotypic missing data <30%) and their corresponding 67 parents.

### Linkage map

**Figure 2:**
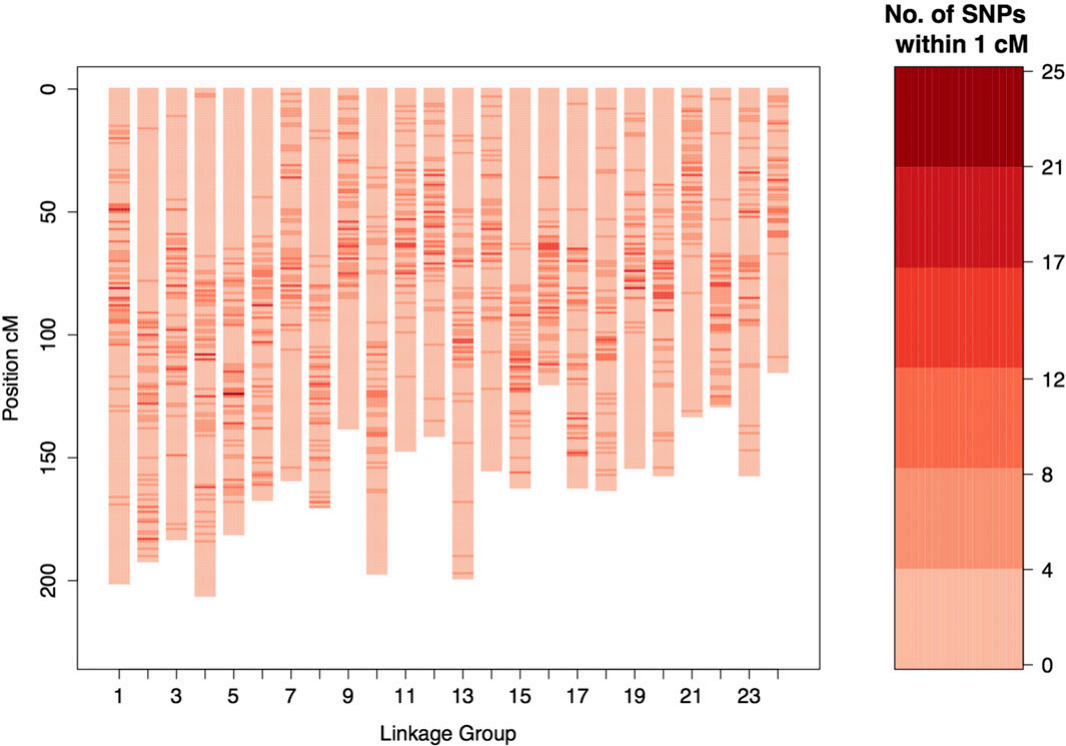
The Gilthead sea bream linkage map. The heatmap on the right side provides scale of color coding for the size of SNP clusters.

The linkage map consisted of 12,085 SNPs that were grouped into 24 linkage groups, in accordance with gilthead sea bream karyotype, with a total map length of 3899 cM (Figure 2, Table 1, and Table S5). The remaining SNPs (9889) either failed to pass QC filters, or were not placed on the resulting linkage groups during mapping, and these were discarded. The female and male maps were comparable with total lengths of 3822 and 4010 cM, respectively. The number of SNPs per chromosome ranged from 366 to 607 (mean = 503; SD = 53), while linkage group length ranged from 115 to 202 cM (mean = 162; SD = 26). The correlation between number of SNPs and corresponding chromosome map length was 0.74 (*n* = 24 linkage groups).

**Table 1:** Consensus linkage map

Linkage Group	No. Markers	Length (cM)
1	607	202
2	602	192
3	558	183
4	560	206
5	554	182
6	536	167
7	530	159
8	522	171
9	521	138
10	518	197
11	508	147
12	499	142
13	500	200
14	489	156
15	485	162
16	484	121
17	480	163
18	470	164
19	471	154
20	461	157
21	455	134
22	454	130
23	452	157
24	366	115
Total	12,085	3899

### Heritability estimation and GWAS

**Figure 3:**
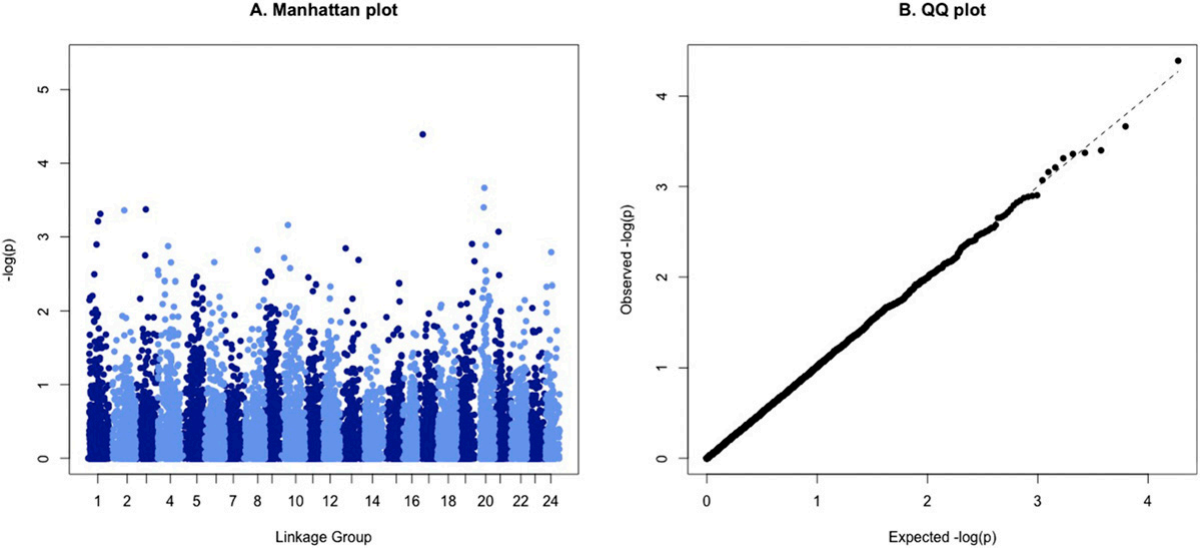
(A) A Manhattan plot highlighting the association between individual SNPs and surviving days. (B) A QQ plot showing the relationship between the observed and expected −log(*P*) values from the GWAS.

The heritability of surviving days (censored data) was 0.22 (95% highest density interval: 0.11–0.36) and 0.28 (95% highest density interval: 0.17–0.4) using the pedigree and the genomic relationship matrix, respectively, of the 777 disease challenged offspring. No SNPs surpassed the Bonferroni-corrected genome-wide significance threshold (*P* = 4.1 × 10^−6^; α = 0.05; Table S6). The SNPs with the lowest *P* values (*P* < 10^−3^) were located in linkage groups 1–3, 10, 17, 20, and 21 (Figure 3).

### Genomic prediction

Genomic prediction was tested as a means of obtaining breeding values, and compared to prediction using a pedigree-based approach (File S1). The prediction was conducted using genotype information from 11,239 SNP markers (passed QC filters set for genomic prediction) for the 777 disease challenged animals that were randomly split in training (*n* = 578) and validation (*n* = 200) datasets. The application of all the genomic prediction models resulted in higher accuracies than those achieved using PBLUP. Prediction accuracy with PBLUP was 0.3 *vs.* 0.38–0.46 for the genomic prediction models, with highest accuracy being observed using the BayesA method (Table 2). Prediction accuracy dropped when more sparse SNP marker datasets were used with the last scenario (utilizing SNPs > 5 cM apart), giving similar accuracies with the ones obtained using PBLUP. In the scenario of utilizing only SNPs located >1 cM apart on the linkage map (2614 SNPs), accuracies ranged between 0.3 and 0.36, with the highest accuracy obtained using rrBLUP. For the dataset utilizing only SNPs located >5 cM apart (705 SNPs), accuracies ranged between 0.29 and 0.31, with highest accuracies obtained using rrBLUP and BayesA.

**Table 2:** Genomic prediction accuracies for surviving days

Model	Accuracy*^a^*	Accuracy	Accuracy
		SNPs 1 cM apart*^b^*	SNPs 5 cM apart*^c^*
PBLUP	0.30 ± 0.03	—	—
rrBLUP	0.44 ± 0.04	0.36 ± 0.03	0.31 ± 0.04
BayesA	0.46 ± 0.03	0.35 ± 0.03	0.31 ± 0.04
BayesB	0.38 ± 0.03	0.30 ± 0.04	0.29 ± 0.03
BayesC	0.44 ± 0.04	0.35 ± 0.04	0.29 ± 0.03

All data are presented as ± SEM.

a Analysis included 12,085 SNPs.

b Analysis included 2614 SNPs.

c Analysis included 705 SNPs.

## Discussion

Gilthead sea bream (*S. aurata*) is a farmed species of paramount importance for Mediterranean aquaculture. While vaccines can offer some protection against pasteurellosis, the low immune competence observed in larval and juvenile stages renders this protection temporary (Antonello *et al.* 2009). Breeding for improved genetic resistance offers an additional and complementary tool to combat losses due to this disease. While traditional family-based selective breeding is applied in sea bream, it cannot utilize within-family genetic variation in the trait. Applying genomic information into selective breeding schemes raises the possibility of selecting directly for favorable alleles at major QTL (marker-assisted selection), or incorporating all markers in the prediction of breeding values (genomic selection). As such, genomics-enabled breeding can expedite the rate of genetic gain, and can potentially reduce the need for yearly trait recording. However, to enable these benefits, substantial genomic resources are typically required (*e.g.*, a high-density SNP genotyping platform), which is currently lacking in sea bream. This is likely to change in the near future as the reference genome sequence and associated genomic tools/data become available. In the meantime, RAD sequencing and similar techniques can readily be applied to generate genome-wide SNP marker datasets, even in the absence of such genomic resources (Baird *et al.* 2008).

High-density SNP linkage maps have been constructed for several aquaculture species, and are useful for both QTL positioning and reference genome assembly (Gonen *et al.* 2014; Palaiokostas *et al.* 2013a). The most recent linkage map of sea bream consists mainly of microsatellites (Tsigenopoulos *et al.* 2014), lacking the necessary resolution for successful implementation of GWAS and genomic prediction. In the current study, we present the first high-density linkage map for this species, consisting of 12,085 SNPs on 24 linkage groups, which is consistent with the karyotype. The genetic map presented here spans 3899 cM, while the map of Tsigenopoulos *et al.* (2014) has a total length of 1769.7 cM, which may reflect the larger number of markers used in the current study. This trend of increase in map distance with increased marker density was observed with previous sea bass (*Dicentrarchus labrax*) linkage maps (Chistiakov *et al.* 2005, 2008; Palaiokostas *et al.* 2015).

The estimated heritability of resistance was moderate (0.22 and 0.28 for the different models) compared to those previously reported for disease resistance traits in various aquaculture species (Ødegård *et al.* 2011). Nonetheless, successful implementation even in the case of low heritability traits in breeding programs is still possible, as demonstrated in livestock (Heringstad *et al.* 2003). Also, since heritability of mortality traits are frequency dependent, with maximal values reported at intermediate mortality levels (Bishop and Woolliams 2014), the low survival rate in the current study may have resulted in an underestimate, and analysis of additional challenge and field data are merited.

The GWAS results pointed to a polygenic or oligogenic genetic architecture for resistance to pasteurellosis, with no genome-wide significant QTL identified, with the lowest *P* values indicative of putative suggestive QTL on linkage groups 1–3, 10, 17, 20, and 21. Unfortunately, the lack of an integrated linkage map for sea bream does not allow direct comparison of the previously described resistance QTL (Massault *et al.* 2010) with the current study. Furthermore no major QTL were identified in the above study, with the largest QTL for surviving days explaining ∼4% of the phenotypic variance. The fact that genomic prediction using all tested models gave similar prediction accuracies, a phenomenon often observed in the study of polygenic traits (Meuwissen *et al.* 2001; Kizilkaya *et al.* 2010), seems to support the hypothesis of polygenic resistance. Nevertheless, since the selected priors will influence the output of the Bayesian models (Gianola 2013), interpretation of genetic architecture based on these results should be treated with caution. Additionally, the moderate sample size in the current study is likely to limit the statistical power to detect small to medium effect QTL. Further, the low mortality level precludes effective estimation of genetic parameters for survival *per se*, and the genetic correlation between survival time (days to death) and overall survival is unknown.

The results from the genomic prediction approach were encouraging for practical implementation of selective breeding for genetic resistance in sea bream, with the genomic prediction models outperforming traditional BLUP. The advantage of the genomic-based models was retained also when only SNPs at 1-cM location intervals were used, which may be useful for reducing genotyping costs and improving cost effectiveness. However, due to the limited number of families in the current study, the training and validation sets contain closely related animals, which will increase the accuracy of prediction. Additional testing of genomic prediction at varying marker densities on separate and preferably larger populations would be required to ascertain the appropriate density for commercial application of genomic selection. The benefit of genomic prediction over a pedigree-based approach is likely because of the ability to capture within-family genetic variation. In mass-spawning species such as sea bream, in which family size and structure is difficult to control, this approach is likely to be particularly advantageous. Overall, the current study demonstrates that SNP markers generated via 2b-RAD are effective at capturing the genetic variation in a complex trait in a sea bream breeding population. This approach is likely to be useful in other species with less-developed genomic tools, and provides further evidence that incorporation of genomic selection is likely to result in significant improvement in selection accuracy and genetic gain compared to traditional family selection in aquaculture breeding.

### Conclusions

2b-RAD sequencing was applied to investigate genetic resistance to pasteurellosis in gilthead sea bream. The SNP data generated were applied to create the first high-density linkage map for sea bream. Only suggestive QTL were detected, implying that resistance to pasteurellosis has an oligogenic-polygenic architecture for the studied population. Genomic prediction using the 2b-RAD genotype data were effective, with substantial improvement in prediction accuracy over the pedigree-based model. This highlights the utility of genotyping by sequencing for genomic prediction of disease resistance in aquaculture species, and its potential to apply genomic selection in commercial breeding programs.

## 

## Supplementary Material

Supplemental Material
