# Supplementary material for: Genomic Prediction of Resistance to Pasteurellosis in Gilthead Sea Bream (Sparus aurata) Using 2b-RAD Sequencing
Source: G3 (Bethesda). 2016 Sep 20;6(11):3693–700. doi: 10.1534/g3.116.035220 (PMC5100868; doi:10.1534/g3.116.035220)
Supplement: Supplemental Material [file supp_6_11_3693__index.html]

Genomic Prediction of Resistance to Pasteurellosis in Gilthead Sea Bream (Sparus aurata) Using 2b-RAD Sequencing — Genomic Prediction of Resistance to Pasteurellosis in Gilthead Sea Bream (Sparus aurata) Using 2b-RAD Sequencing — Supplemental Material 

# Genomic Prediction of Resistance to Pasteurellosis in Gilthead Sea Bream (*Sparus aurata*) Using 2b-RAD Sequencing

## Supplemental Material for Palaiokostas *et al.*, 2016

**Files in this Data Supplement:**

- Table S1 - Samples phenotypes. (.txt, 12 KB)
- Table S2 - Pedigree. (.txt, 18 KB)
- Table S3 - Parental raw read output. (.txt, 2 KB)
- Table S4 - Offspring raw read output. (.txt, 24 KB)
- Table S5 - Genetic map. (.txt, 208 KB)
- Table S6 - Genotypic data. (.txt, 18 MB)
- File S1 - Genomic prediction. (.zip, 2 KB)
